# History of a Case of Fungous Exostosis Affecting the Scapula

**Published:** 1821-08

**Authors:** Edw. Th. Bond

**Affiliations:** Member of the Royal College of Surgeons.


### *History of a Case of Fungous Exostosis affecting the Scapula.* By

EDW. TH. BOND, Member of the Royal College of Surgeons.

ABOUT the middle of August, 1820, I visited Thomas Gammidge, a farmer resident in the neighbourhood of Nun-Eaton, a stout man of six feet two, and of a frame proportioned to his height. His constitution was originally robust, and his health, until within some few previous months, had been generally good; but, at the period of my first seeing him, derangement of the digestive organs had been for some time a subject of complaint. The immediate cause of my attendance was an injury he had received on the leg; the direct consequences of which were soon removed by the means used; but œdema supervened in the injured limb, in proportion as inflammation disappeared. Constitutional disorder became a more prominent source of distress, and the symptoms denoting feeble stomach and impaired digestion did not give way to the regimen and medicines which are usually efficacious. On my fourth or fifth visit, when about to take my leave, he mentioned that he had been for some time annoyed by a rheumatic pain in the right shoulder, and, on my request to examine the part, referred his complaints to the space between the spine of the scapula and its inferior angle. The pain was far from being so urgent as to occasion him anxiety, and probably, but for my being then present, would for a time have passed unnoticed. It was not aggravated by pressure; there was no discoloration of the integuments; no swelling; and his pulse was not more than sixty. He informed me that, in the spring of the year, the struggles of a calf which he was leading had caused him to fall with the front of his shoulder against a door-post, and that the bruise gave him at the moment considerable pain; yet, as some intermediate time had passed in perfect ease, he was unwilling to believe that there existed any connexion, as cause and effect, between such injury and his present symptoms; and, though he assured me that he had never experienced any previous uneasiness in the part, appeared by no means inclined to trace the existing affection to so distant an origin.

From this time I saw him no more for some months. His health, however, became progressively worse, and the disease in the shoulder gradually assumed a more and more formidable aspect. He suffered much from severe local pain and consequent general irritation; nor was it long before a tumor made its appearance in the part to which he had originally referred his uneasiness. After a short time this advanced rapidly in size; and, in February 1821, in consequence of another surgeon having proposed an operation for its removal, my attendance was again requested to give an opinion respecting the propriety of the measure. At this date it would have weighed not less than six or seven pounds. It appeared in a degree moveable. The spine of the scapula could be distinguished through the integuments, and the tumor seemed to have had its origin immediately below this process; in its descent from which it appeared as though it had turned upwards under the inferior angle and lower portion of the basis, so as to elevate the whole scapular division of the shoulder. Its chief bulk was situated upon the dorsum of the bone, but it reached in front as far as the dorsal margin of the axilla.

I did not at this time entertain any suspicion of its true and most formidable nature; and therefore, having first proposed a farther consultation, coincided in recommending the knife: but this advice was eventually negatived by the concurrent opinions of my father, who considered that the operation would be attended with too much risk, and of two other practitioners, who regarded the tumor as no more than a deep-seated abscess. This latter conjecture afforded the patient hope, without calling upon him for any exertion of mind; and, with the futile anticipations of the falling man who dreams not that the bough at which he catches may be broken, he clung to the belief that the whole mass would suppurate and be dissipated. Under this impression, its rapidly-increasing bulk and severer pain were not, in his eye, so much the heralds of approaching dissolution, as the harbingers of no very distant convalescence; and he continued sinking towards the grave, in the delusive expectation that the time was drawing near for his recovery.

I inspected the tumor about ten days before his death. It had increased with great rapidity in every direction, but particularly at its upper part, and was at least a full half larger than at the period last mentioned. It was no longer moveable. The spine of the scapula could not now be felt through the integuments; and these were uneven, of irregular thickness, and in places shining. Over the greater portion of the tumor they were firm and unyielding under pressure; but here and there they were softer and more elastic to the touch, conveying a sensation as though a fluid existed underneath.

He died on the 4th of April, and the following are the results of an examination which took place on the fourth subsequent day. The inferior portion of the tumor had insinuated itself to a considerable distance beneath the latissimus dorsi, which was pale and flabby. Towards its upper extremity, the trapezius, at its usual point of insertion into the spine of the scapula, was intimately blended with the diseased substance, and had lost in great part the appearance of muscle. Indeed, it rather resembled dark-coloured liver, or a very thick and dense stratum of coagulated blood. On the surface of the tumor was a very firm fascia-like expansion, which nevertheless became gradually thinner as it approached the lower part, and was probably no more than what had in health covered the subjacent supra and infra spinati muscles; but it had lost its original glossiness, and was now of a dull-brownish hue. The fleshy mass which was exposed on the removal of this fascia had a soft, spongy, elastic feel, and somewhat of a variegated appearance; its colour partaking in some places of an ashy tinge, while in others it was of a dusky unhealthy red. This was more particularly the case at its inferior extremity, as it drew to a point under the latissimus dorsi, where, except in hue, it was not very unlike convolutions of brain, and so soft that it was divided unawares. Towards the middle of its surface, spiculæ of bone presented themselves, and the disease, on being cut into, exhibited appearances which none who had seen the patient during life had anticipated. A morbid growth of bone extended nearly through the whole, and not only was there an exuberant formation of new osseous matter, but, in addition, a complete change in the structure of the original bone. No vestige remained of even its form, but in its stead there was a diseased osseous mass, of a spongy texture, which, in every direction, and through its entire diameter, yielded readily to the scalpel. Small cells, apparently isolated, containing a straw-coloured fluid, were irregularly interspersed through the substance of the tumor; and towards its centre were four or five ounces of a dark grumous fluid, of the consistence of pus, the sides of the cavity containing which bore marks of high vascularity, and were irregular, ragged, and undefined, with coagula of blood adhering to them. The supra spinatus, infra spinatus, and subscapularis, seemed to have been converted into what resembled adipose substance; for there were several corresponding portions of the disease having this appearance, and no other traces of these muscles were observed. The tumor felt greasy when handled, and was in close apposition with the ribs, but the hand could be readily passed between the two. The cavity of the joint was not examined.

There are one or two considerations which a perusal of the case is calculated peculiarly to excite. It impresses on the mind the prudence of making a guarded prognosis, and raises an interest as to the probable consequences of an operation, if such had been undertaken. It is to be presumed that the surgeon would only have become acquainted with the disease of the bone when his hands were already bloody; and, under these circumstances, the preferable turn of the dilemma would have been the removal of the scapula itself. In the present instance, the morbid condition of bony structure was co-existent with, so far as we know, an incurable affection of the soft parts; but cases may offer themselves without any such formidable coincidence, and in such I see no equivalent objection to the operation of which I am speaking. I do not say that it is one which a surgeon would adopt from preference; but, under certain circumstances, it may be the only practice in our power, and, in fact, the instances are but few where we have before us a choice of measures promising equal results.

*Nun-Eaton; June 8th*, 1821.

